# The influence of distance weight on the inverse distance weighted method for ore-grade estimation

**DOI:** 10.1038/s41598-021-82227-y

**Published:** 2021-01-29

**Authors:** Zhan-Ning Liu, Xiao-Yan Yu, Li-Feng Jia, Yuan-Sheng Wang, Yu-Chen Song, Hai-Dong Meng

**Affiliations:** 1 Anyang Institute of Technology, Anyang, 455000 People’s Republic of China; 2 Inner Mongolia University of Science and Technology, Baotou, 014010 People’s Republic of China

**Keywords:** Mineralogy, Applied mathematics, Statistics

## Abstract

In order to study the influence of distance weight on ore-grade estimation, the inverse distance weighted (IDW) is used to estimate the Ni grade and MgO grade of serpentinite ore based on a three-dimensional ore body model and related block models. Manhattan distance, Euclidean distance, Chebyshev distance, and multiple forms of the Minkowski distance are used to calculate distance weight of IDW. Results show that using the Minkowski distance for the distance weight calculation is feasible. The law of the estimated results along with the distance weight is given. The study expands the distance weight calculation method in the IDW method, and a new method for improving estimation accuracy is given. Researchers can choose different weight calculation methods according to their needs. In this study, the estimated effect is best when the power of the Minkowski distance is 3 for a 10 m × 10 m × 10 m block model. For a 20 m × 20 m × 20 m block model, the estimated effect is best when the power of the Minkowski distance is 9.

## Introduction

The inverse distance weighted (IDW) method as an interpolation method^1^. Widely used in, image interpolation^2^, spatial data interpolation^3,4^, and algorithm optimization^5,6^. The IDW method is considered to be a highly adaptable resource estimation method^7^. Studies focusing on the IDW method as a resource estimation tool focus on two aspects: estimating natural recourses and improving the IDW method. The IDW method has been used to estimate grade^8,9^, such as Cu and Mo^10^. On the other hand, a significant amount of research has been done on improving the IDW method. The weight coefficient distribution was refined, and the estimation accuracy was improved by modifying the weight of the angle weight coefficient^11^. The IDW method was further improved to consider directionality and extrapolate data^12^. Researchers proposed the Indicated-IDW, which reported and improved estimation accuracy^13^. The improvements reflect the anisotropic characteristics of ore grade; however, its application scope is limited.

The IDW method is a biased estimation method^14^; however, there is little research on the systematic deviation of IDW estimation. The authors estimate Ni and MgO grades of serpentinite ore with the starting of the above question, introduce Minkowski distance as the distance weight of IDW and examine the effect of distance weight and grade distribution on estimation deviation. The distance weight calculation method is extended, and system estimation grade deviations for different distance weights are given.

## Data sources and research methods

### Overview of the study area

The study area is located on the southern margin of the Sino-Mongolian trough. The NE-trending and NW-trending structures in the area constitute the overall tectonic framework of the mining area. The rock type is basic-ultrabasic, with intrusive rocks being the most common rock type. Serpentinite ore bodies occur in the Carboniferous Benbantu Formation (C_2_bb) tuffaceous slate, specifically the No. 1 and No. 2 ore bodies. In this study, the No. 1 ore body was used for example. The No. 1 serpentinite ore body has the length of 1166 m, a maximum control depth of 590 m, a strike of 108°, the inclination angle is 198°, an inclination angle of 9–26°, and average inclination angle of 17°.The true thickness of ore body is between 26.53 and 145.19 m, with an average thickness of 91.47 m. Thickness changing coefficient of the ore body is 45%.

### Data collection and processing

**Figure 1:**
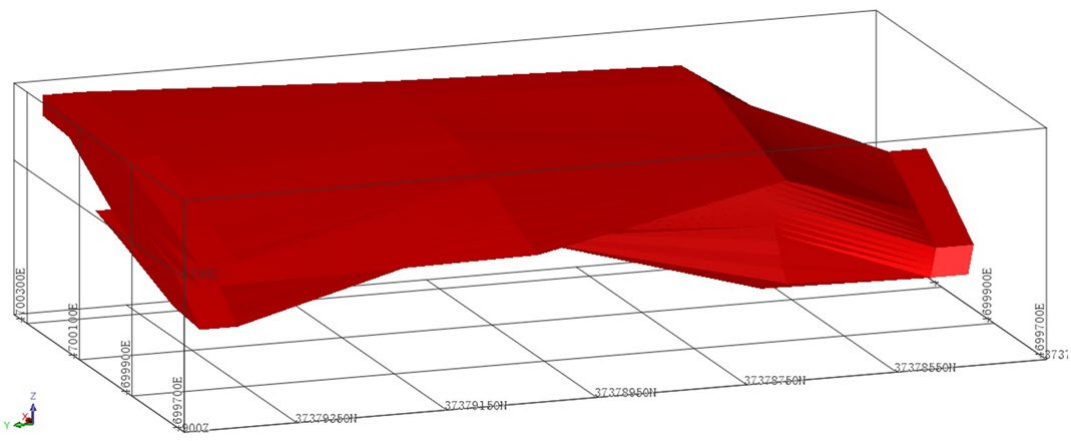
3D model of ore body.

1. Ore body model and block model
  Serpentine ore physical parameters are used to build a three-dimensional model of the ore body (Fig. 1), and two types of corresponding block models. Model 1 has a block size of 10 m × 10 m × 10 m and contains 50,638 blocks. Model 2 has a block size of 20 m × 20 m × 20 m and contains 7532 blocks (Fig. 2).

**Figure 2:**
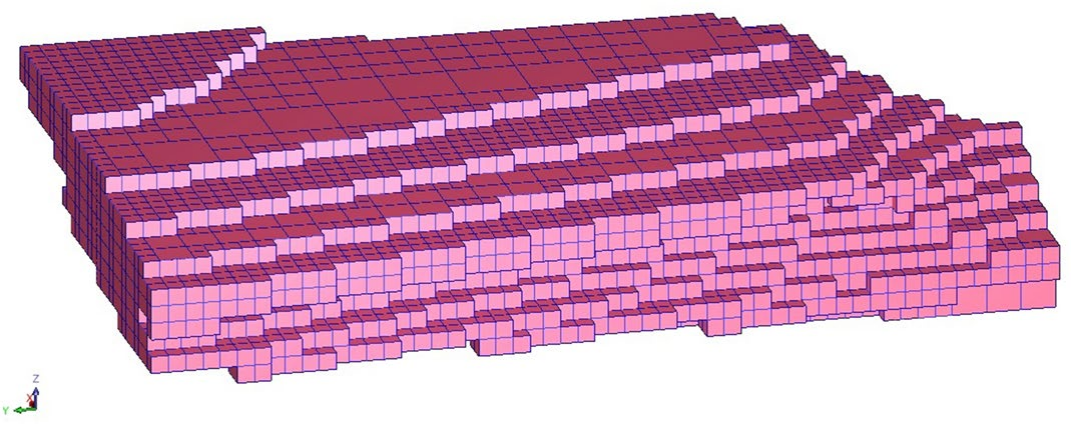
Block model.
2. Sample grade statistics
  188 serpentinite ore samples were subjected to ore-grade estimation. Samples were collected in past exploration projects. Figures 3 and 4 show the Ni and MgO grade histograms respectively.

**Figure 3:**
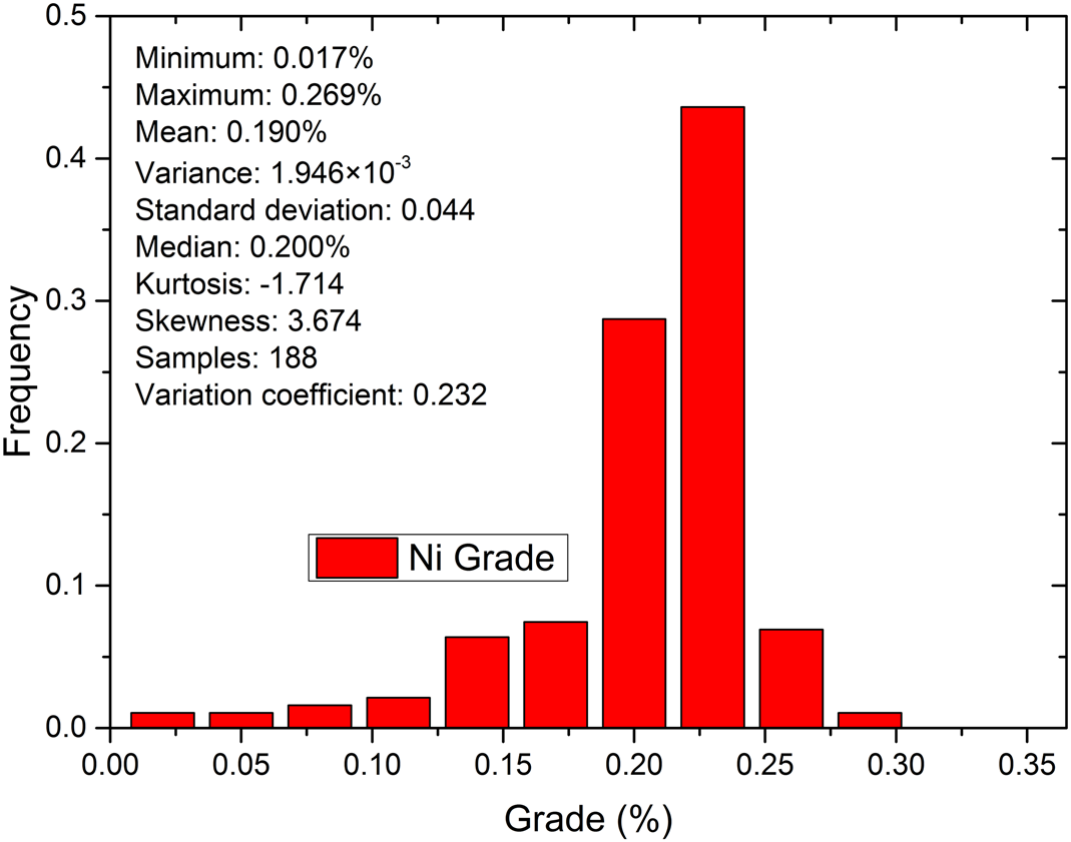
Grade distribution of Ni samples.

**Figure 4:**
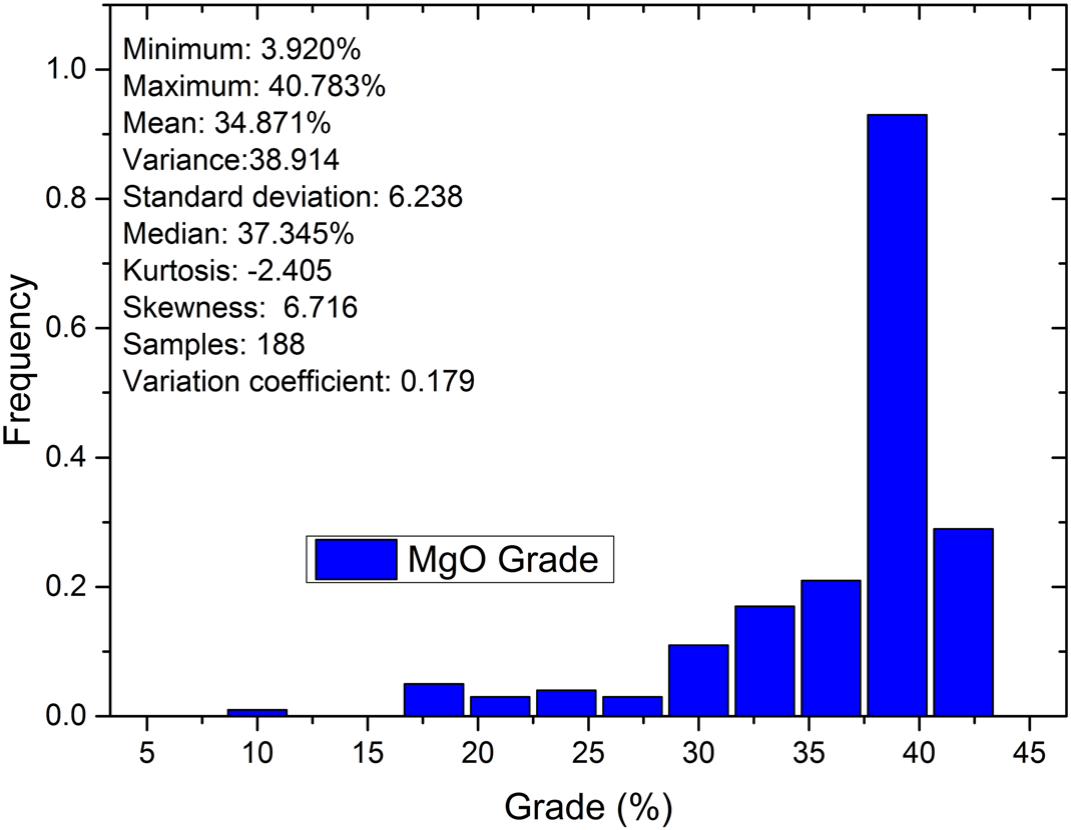
Grade distribution of MgO samples.

### Research methodology

The IDW method is a spatial interpolation method^15^, which uses spatial distance for the correlation calculation that is the distance weight calculation. For an unknown point *P* with position (*x*_*0*_, *y*_*0*_, *z*_*0*_), there are known points around it. Assuming that each known point has spatial coordinates of (*x*_*i*_, *y*_*i*_, *z*_*i*_) (*i* = 1, 2, …, *n*), and the attribute value is *P*_*i*_. The distance between each known point and unknown point is *d*_*i*_(*x*, *y*, *z*). IDW is used to estimate the property value of each unknown point. Equation (1) represents this estimation^16^. In Eq. (1), *m* is the power in the inverse distance power law.

1 $$ P = \sum\limits_{i = 1}^{n} {{{\frac{{P_{i} }}{{[d_{i} (x,y,z)]^{m} }}} \mathord{\left/ {\vphantom {{\frac{{P_{i} }}{{[d_{i} (x,y,z)]^{m} }}} {\sum\limits_{i = 1}^{n} {\frac{1}{{[d_{i} (x,y,z)]^{m} }}} }}} \right. \kern-\nulldelimiterspace} {\sum\limits_{i = 1}^{n} {\frac{1}{{[d_{i} (x,y,z)]^{m} }}} }}} $$

In the study, the special form of Minkowski distance^17^ is used in the distance weight calculation method of IDW. Minkowski distance is expressed as:

2 $$ d_{i} = \sqrt[p]{{\left| {x_{0} - x_{i} } \right|^{p} + \left| {y_{0} - y_{i} } \right|^{p} + \left| {z_{0} - z_{i} } \right|^{p} }} $$

where *p* is the power of the Minkowski distance, and *p* can be 0 to + ∞. When *p* = 1, it is the Manhattan distance^18,19^. It is expressed as:

3 $$ d_{i} = \left| {x_{0} - x_{i} } \right| + \left| {y_{0} - y_{i} } \right| + \left| {z_{0} - z_{i} } \right| $$

When *p* = 2, it is Euclidean distance. It is expressed as:

4 $$ d_{i} = \sqrt[{}]{{\left( {x_{0} - x_{i} } \right)^{2} + \left( {y_{0} - y_{i} } \right)^{2} + \left( {z_{0} - z_{i} } \right)^{2} }} $$

When *p* →  + ∞, it is Chebyshev distance. It is expressed as:

5 $$ d_{i} = \mathop {{\text{lim}}}\limits_{p \to \infty } \left( {\left| {x_{0} - x_{i} } \right|^{p} + \left| {y_{0} - y_{i} } \right|^{p} + \left| {z_{0} - z_{i} } \right|^{p} } \right)^{{{1 \mathord{\left/ {\vphantom {1 p}} \right. \kern-\nulldelimiterspace} p}}} $$

The study also involves a number of sample points (*n* in Eq. (1), the value is 3), the distance power (*m* in Eq. (1), value is 2), and search radius (value is 300* m*). Estimation deviation is a method of comparing estimated grade to sample grade. The deviation is calculated as: deviation = (estimation grade − sample grade)/sample grade × 100%.

### Results and analysis

Estimated grades of Ni and MgO are given in Tables 1 and 2.

**Table 1:** Estimation grade statistics of model 1.

Distance type	Grade name	Minkowski distance										
		Manhattan (p = 1)	Euclidean (p = 2)	p = 3	p = 5	p = 7	p = 9	p = 11	p = 13	p = 15	p = 20	Chebyshev p → ∞
Minimum	Ni	0.020	0.056	0.056	0.056	0.056	0.056	0.056	0.056	0.056	0.056	0.056
	MgO	8.83	16.12	17.14	13.92	17.17	17.19	16.90	16.90	16.90	16.90	16.90
Maximum	Ni	0.263	0.263	0.263	0.263	0.263	0.263	0.263	0.263	0.263	0.263	0.263
	MgO	40.14	40.14	40.14	40.14	40.14	40.14	40.14	40.14	40.14	40.14	40.14
Mean	Ni	0.182	0.183	0.183	0.183	0.182	0.182	0.182	0.183	0.184	0.186	0.194
	MgO	33.40	33.79	33.83	33.79	33.77	33.74	33.74	33.89	34.04	34.36	35.22
Variance	Ni	0.0013	0.0014	0.0015	0.0015	0.0015	0.0015	0.0015	0.0015	0.0014	0.0013	0.0008
	MgO	35.807	32.368	32.699	32.930	32.973	33.096	32.899	31.053	29.104	25.550	15.200
Standard deviation	Ni	0.036	0.038	0.038	0.039	0.039	0.039	0.039	0.039	0.038	0.036	0.029
	MgO	5.984	5.689	5.718	5.739	5.742	5.753	5.736	5.573	5.395	5.055	3.899
Medan	Ni	0.1884	0.192	0.192	0.192	0.192	0.192	0.1903	0.1933	0.1936	0.1941	0.2028
	MgO	35.58	35.80	35.85	35.80	35.80	35.80	35.80	35.81	35.85	35.89	37.09
Kurtosis	Ni	− 1.293	− 1.295	− 1.288	− 1.276	− 1.273	− 1.267	− 1.264	− 1.293	− 1.316	− 1.357	− 0.782
	MgO	− 1.583	− 1.416	− 1.419	− 1.412	− 1.408	− 1.403	− 1.403	− 1.462	− 1.502	− 1.584	− 1.193
Skewness	Ni	2.245	1.942	1.874	1.818	1.801	1.764	1.742	1.876	2.035	2.431	0.303
	MgO	2.444	1.311	1.281	1.250	1.236	1.217	1.238	1.542	1.815	2.401	1.390
Variation coefficient	Ni	0.198	0.208	0.210	0.211	0.212	0.213	0.214	0.211	0.206	0.195	0.148
	MgO	0.179	0.168	0.169	0.170	0.170	0.171	0.170	0.164	0.159	0.147	0.111

**Table 2:** Estimation grade statistics of model 2.

Variables	Grade name	Minkowski distance										
		Manhattan (p = 1)	Euclidean (p = 2)	p = 3	p = 5	p = 7	p = 9	p = 11	p = 13	p = 15	p = 20	Chebyshev p → ∞
Minimum	Ni	0.017	0.020	0.020	0.038	0.039	0.059	0.052	0.059	0.059	0.059	0.059
	MgO	3.920	7.976	8.136	8.566	8.570	8.573	8.573	8.573	8.573	8.573	8.573
Maximum	Ni	0.257	0.257	0.257	0.257	0.257	0.257	0.257	0.257	0.263	0.263	0.263
	MgO	40.250	40.417	40.250	39.614	39.609	39.603	39.601	39.601	39.615	39.615	39.601
Mean	Ni	0.155	0.173	0.170	0.171	0.170	0.175	0.173	0.173	0.175	0.176	0.179
	MgO	31.091	32.212	32.057	32.075	31.874	32.352	32.172	32.342	32.565	32.810	33.182
Variance	Ni	0.003	0.002	0.002	0.002	0.002	0.002	0.002	0.002	0.002	0.002	0.001
	MgO	52.545	48.470	54.071	53.204	52.451	45.631	44.587	42.934	41.457	39.329	32.492
Standard deviation	Ni	0.057	0.044	0.049	0.048	0.049	0.042	0.042	0.042	0.042	0.041	0.038
	MgO	7.249	6.962	7.353	7.294	7.242	6.755	6.677	6.552	6.439	6.271	5.700
Medan	Ni	0.177	0.183	0.186	0.186	0.183	0.183	0.183	0.183	0.186	0.188	0.188
	MgO	32.848	34.748	35.239	35.173	34.568	34.396	34.036	34.036	34.396	34.413	33.534
Kurtosis	Ni	− 0.813	− 1.060	− 1.067	− 1.060	− 1.076	− 0.960	− 0.832	− 0.846	− 0.884	− 0.864	− 0.547
	MgO	− 0.931	− 1.297	− 1.248	− 1.222	− 1.133	− 1.341	− 1.247	− 1.301	− 1.386	− 1.448	− 1.393
Skewness	Ni	− 0.570	0.626	0.377	0.334	0.306	0.501	0.165	0.230	0.251	0.227	− 0.777
	MgO	0.044	1.163	0.772	0.682	0.451	1.370	1.145	1.410	1.745	2.107	2.792
Variation coefficient	Ni	0.367	0.256	0.290	0.283	0.291	0.238	0.244	0.242	0.241	0.235	0.212
	MgO	0.233	0.216	0.229	0.227	0.227	0.209	0.208	0.203	0.198	0.191	0.172

**Figure 5:**
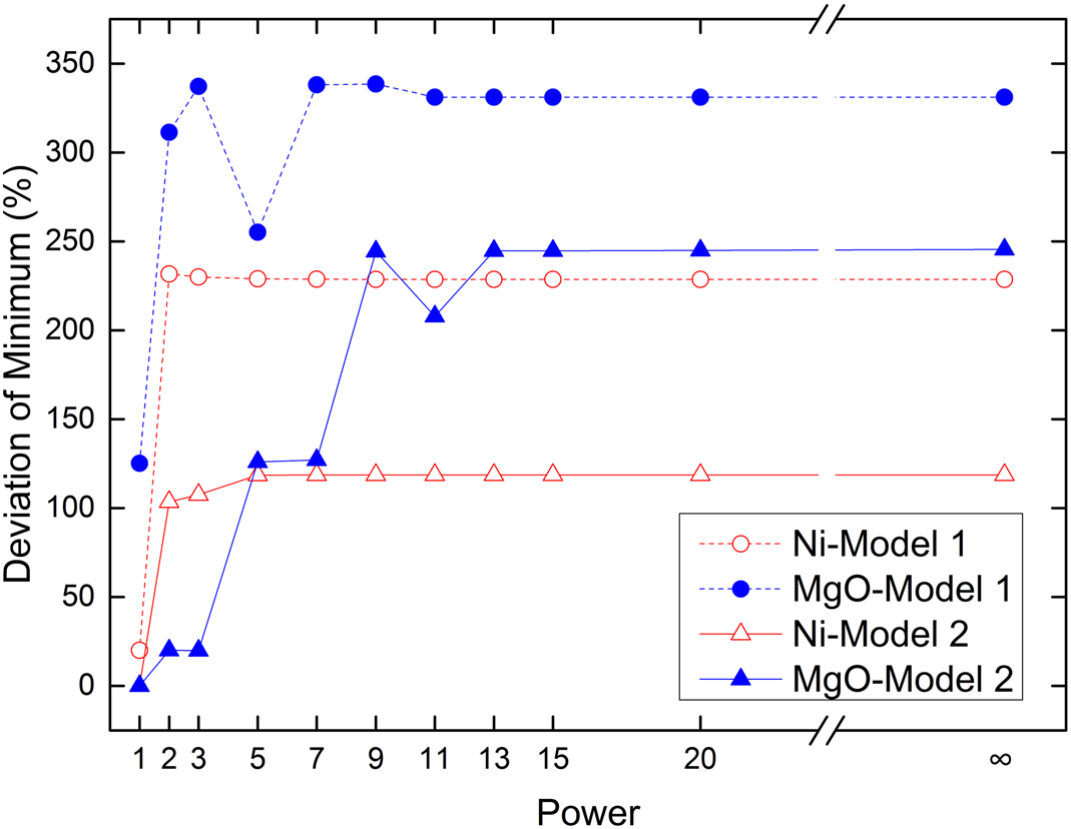
Deviation of minimum ore grade.

**Figure 6:**
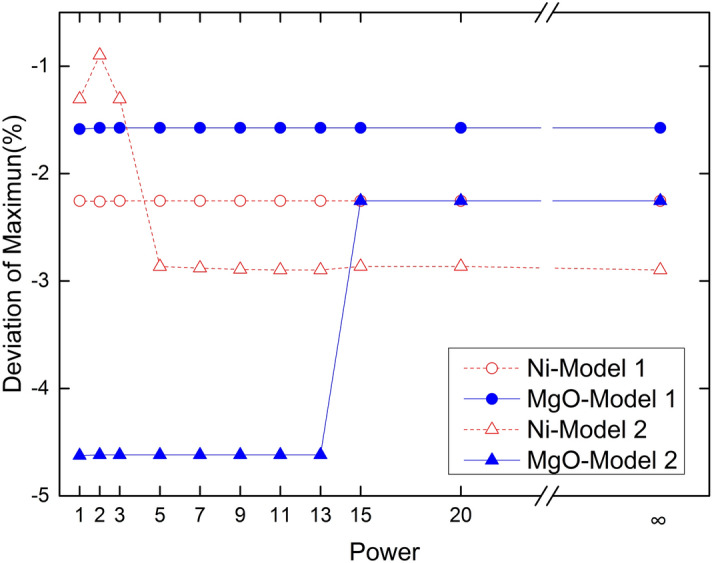
Deviation of maximum ore grade.

**Figure 7:**
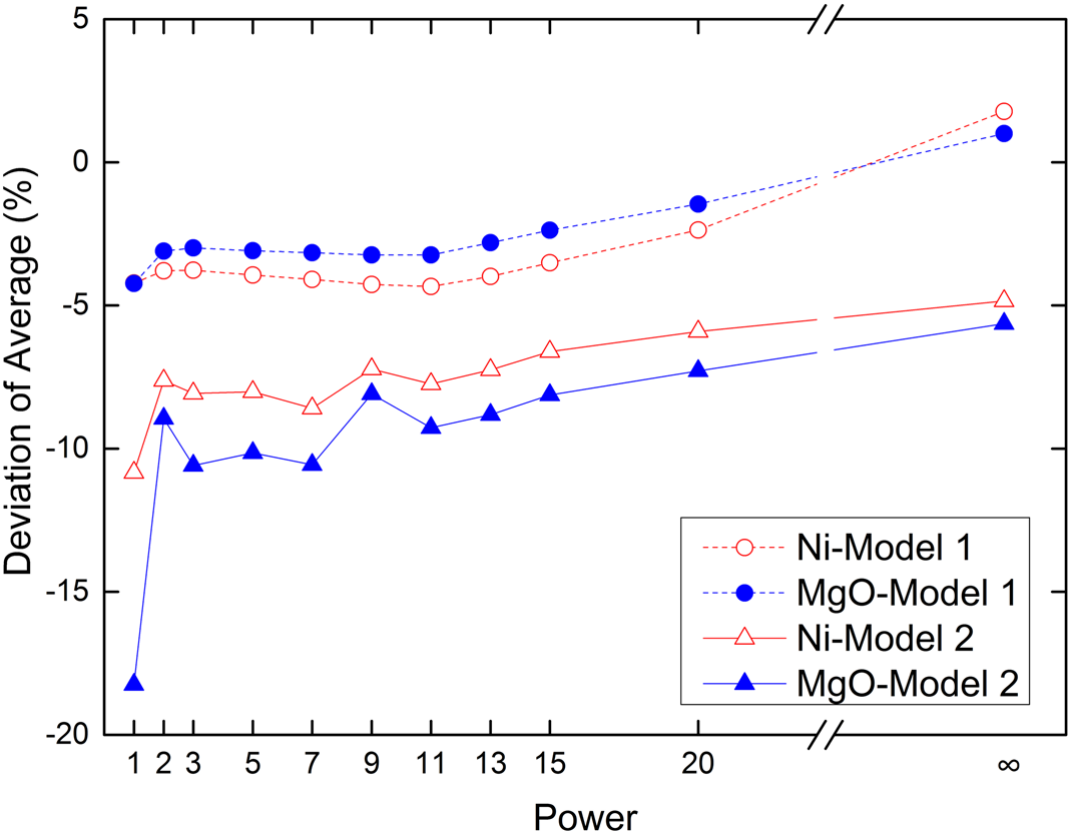
Deviation of average ore grade.

**Figure 8:**
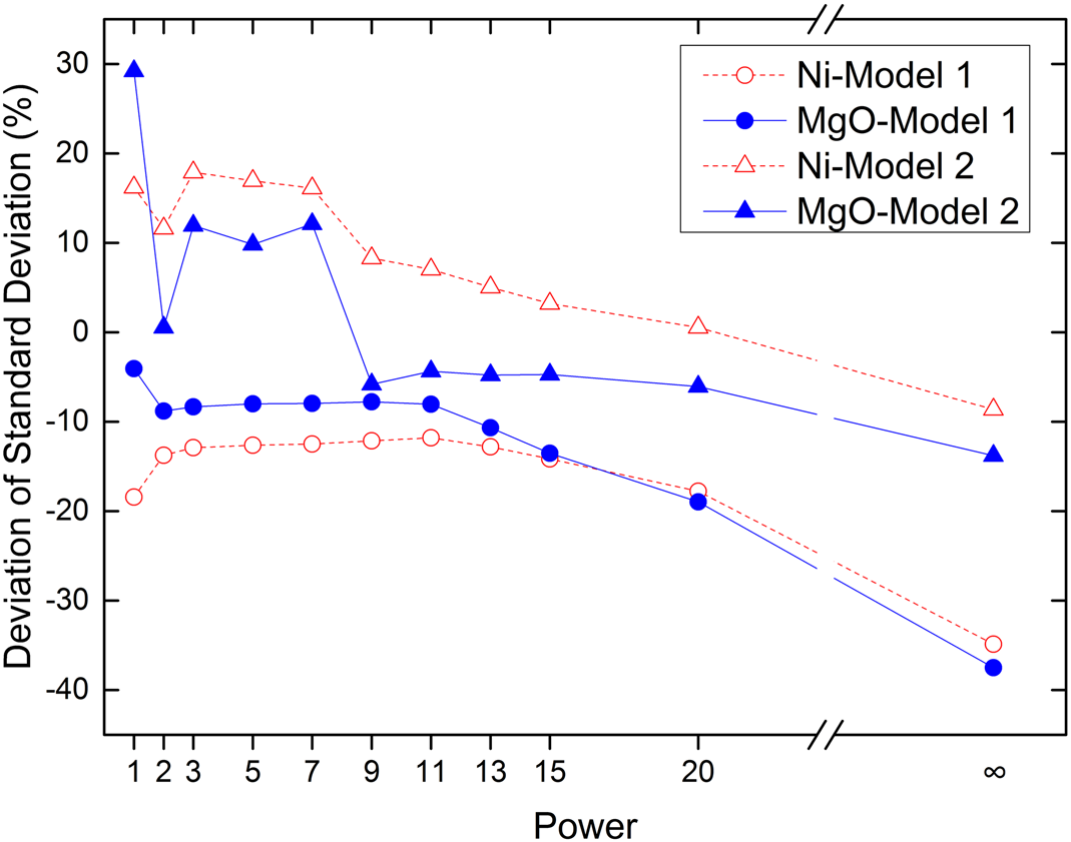
Deviation of ore grade standard deviation.

The estimated grade and sample grade are compared. Figures 5, 6, 7, and 8 represent the estimated deviation in minimum, maximum, average, and standard deviations. The horizontal axis corresponds to Manhattan distance (*p* = 1), Euclidean distance (*p* = 2), Minkowski distance (*p* = 3, 5, 7, 9, 11, 13, 15), and Chebyshev distance (*p* → ∞) (Figs. 5, 6, 7, 8).

The minimum deviation of Ni is less than the minimum deviation of MgO (Fig. 5). Grade distribution and model type have an effect on the estimated IDW results. The sample proportion is only 12.23% with the sample Ni grade between 0.017 and 0.140%. The sample proportion is only 7.45% with MgO grade between 3.92 and 24%.Three samples are used to estimate an unknown point in the estimate. The probability of 3 samples simultaneously being low grade is small, resulting in a larger minimum valuation deviation. Therefore, the deviation of the minimum value is larger. Since the proportion of Ni is larger than that of MgO in the low-grade section, the deviation of MgO grade minimum is greater than that of the Ni grade. The estimated grade minimum deviation from Model 1 is larger than that of Model 2. This may be due to fewer number of samples in the area where the sampled value is lower. The low-grade sample control area is large.

The influence of distance weight on the maximum grade deviation is small (Fig. 6).The maximum deviation of the Ni and MgO estimated grades is negative, indicating that the maximum estimated Ni and MgO grades is less than the maximum sample grade. The Ni maximum grade deviation is greater than that of MgO maximum grade in model 1. Ni grade maximum deviation varies greatly in Manhattan distance, Euclidean distance, and Minkowski distance (p = 3–5) within model 2. Minkowski distance maximum deviation (p = 5–∞) is relatively stable at approximately 2.89%. MgO grade maximum deviation jumps at p = 13–15 and ranges in absolute value from large to small. The maximum deviation after the change is close to Ni in model 1. Therefore, the sample grade distribution and model type has an influence on the estimated results. The lower grade maximum deviations are due to the higher number of high-grade samples, which is the opposite a deviation in minimum grade.

The Ni and MgO grade average deviations clearly form two groups according to model 1 and model 2 (Fig. 7). The average estimated grade is less than the sample grade, when the Minkowski distance power is less than 20 (*p* < 20) in model 1. When Chebyshev distance is used, the average estimated grade is more than the sample grade in model 1. The average estimated grade is less than the sample grade in model 2. The average MgO grade deviation is slightly less than the average Ni grade deviation in models 1 and 2, showing that the estimated deviations for the IDW method are related to the calculation method, distance weight, and sample distribution. The average deviation of model 1 is less than model 2, indicating that block size has an influence on the estimate results and a greater block size bring about a greater average deviation. When Minkowski distance is used to calculate distance weights and *p* < 20, Ni and MgO grades are at risk of being under estimated within model 1. When Chebyshev distance is used, Ni and MgO are at risk of being over estimated. Ni and MgO grades are at risk of being under estimated within model 2.

Deviations in the standard deviation of Ni and MgO grades are presented in two groups according to model 1 and model 2 (Fig. 8). The trend of the deviation in standard deviation is exactly the opposite of the average deviation trend. When the Minkowski distance power is less than 11 (p < 11), deviations in standard deviation are stable in model 1 and are relatively unstable in model 2. When the Minkowski distance power is greater than 11, deviations in standard deviation rapidly increase in model 1 and change from positive to negative in model 2, which is consistent with the trend of model 2. Therefore, deviations in the standard deviation of Ni grade and MgO grade are different, showing that estimated results of the IDW method are affected by the distance weight calculation method and grade distribution. In addition, the trend of the deviation in standard deviation of Ni and MgO is consistent within a given model, indicating that block size has a significant impact on the estimate results.

A comprehensive analysis of the estimated deviation indicates that the estimated ore grade results are regular in the IDW method which uses the Minkowski distances to calculate weights. The effect of the distance weight calculation method on the IDW method is confirmed by the two models. The effect of block size on the estimated result is also significant. The estimation is best in model 1when p = 3.At this time, the average Ni grade deviation is − 3.77%, and the average MgO grade deviation is − 2.99%. The estimation is best in model 2 when p = 9. At this time, the average Ni grade deviation is − 7.22%, and the average MgO grade deviation is − 8.09%.

## Discussion

The IDW method is widely used as a highly adaptive grade estimation method. Previous research seldom examined the distance weight calculation method and estimated system deviation of the IDW method. Estimated grade deviations show that Euclidean distance is not the best option for distance weight calculations; therefore, it is necessary to study the influence of other distance weight calculation methods.

Ni and MgO samples are from the same exploration project, and all samples were collected in the same way; however, when the same distance weight is used, the estimated deviations are not the same. Hence ore grade distribution must have an influence on the estimated results. The variation in the two types of estimated grade deviations is consistent, indicating that the estimated results of IDW are stable for different distance weights. Two model sizes are used to verify the effect of the distance weight calculation method on Ni and MgO grade estimated results. The IDW estimated results of Ni and MgO grade are also divided into two groups according to the two models. The deviations in average and standard deviation of Ni and MgO grade and are consistent for the same block model, which further clarifies the effect of distance weights on IDW estimated results.

This study theoretically expands the calculation method for the distance weight of the IDW method. The Euclidean distance weight is extended to the use of Minkowski distance weight, and a new optional parameter (distance weight) is added. The deviations of the estimated system are given when the Minkowski distance weight is used. This new method can improve ore-grade estimation by choosing the Minkowski distance power value. More study is needed to further verify the effect of the distance weight calculation method on ore-grade estimation, especially the influence of distance weight and grade distribution in the IDW method.

## Conclusion

1. The estimated effect of the Ni and MgO grades is clearly presented in two groups according to block models based on IDW method along with the distance weight. The Ni and MgO grade estimation deviations have high consistency for the same block model, indicating that the distance weight calculation method has a significant impact on IDW. Estimated Ni and MgO grades in serpentinite show that it is feasible to use Minkowski distance for the distance weighting in the IDW method. The study expands the calculation method for distance weight using IDW and gives the variation rule for the estimated Ni and MgO grade deviation with Minkowski distance. The law of the estimated results along with the distance weight is given.
2. This study expands the calculation method for the distance weight in the IDW method and provides a new way to improve estimation accuracy by choosing the distance weight calculation method. Ore-grade estimation of serpentinite deposits shows that the Minkowski distance provides the best estimation when a power of 3 is used within model 1. At this time, the Ni and MgO grade average deviations are − 3.77% and − 2.99%, respectively. The Minkowski distance provides the best Ni and MgO grade estimation when a power of 9 is used within model 2. At this time, the average deviations are − 7.22% and − 8.09%, respectively.
